# Transgenic Mouse Expressing Optical MicroRNA Reporter for Monitoring MicroRNA-124 Action during Development

**DOI:** 10.3389/fnmol.2016.00052

**Published:** 2016-07-12

**Authors:** Yoori Choi, Do won Hwang, Mee Young Kim, Joo Yeon Kim, Woong Sun, Dong Soo Lee

**Affiliations:** ^1^Department of Nuclear Medicine, College of Medicine, Seoul National UniversitySeoul, South Korea; ^2^Department of Molecular Medicine and Biopharmaceutical Sciences, Graduate School of Convergence Science and Technology, and College of Medicine or College of Pharmacy, Seoul National UniversitySeoul, South Korea; ^3^Department of Anatomy, Brain Korea 21, College of Medicine, Korea UniversitySeoul, South Korea

**Keywords:** mir-124, transgenic mouse, bioluminescence imaging, development, neurogenesis

## Abstract

MicroRNAs (miRNAs) fine-tune target protein synthesis by suppressing gene expression, temporally changing along development and possibly in pathological conditions. A method to monitor the action of miRNAs *in vivo* shall help understand their dynamic behavior during development. In this study, we established a transgenic mouse harboring miR-124 responsive element in their luciferase-eGFP reporter transgenes which enabled monitoring the action of miR-124 in the brain and other organs *in vivo* by the bioluminescence imaging. The mouse model was produced and verified by imaging *ex vivo* so that luminescence by luciferase shone and then reduced during development with miR-124 expression. Bioluminescence dramatically decreased in the brain between embryonic day 13 and 16 as endogenous miR-124 expression increased, which sustained into adulthood. The inverse relationship of miR-124 expression was observed with luciferase bioluminescence and activity *ex vivo* as well as *in vivo*. Taken together, one can use this microRNA-transgenic mouse to investigate the temporal changes of microRNA action *in vivo* in the brain as well as in other organs.

## Introduction

MicroRNAs (miRNAs), small endogenous non-coding RNAs of ∼22 nucleotides, regulate gene expression post-transcriptionally in almost all types of cells of living organisms. MiRNAs are transcribed by RNA polymerase II, which yields primary miRNA (pri-miRNA) in the nucleus (Lee et al., 2004). The pri-mRNA is cut in its core by Drosha (endonuclease RNase III), producing a 60∼70 nucleotide-long pre-miRNA (Lee et al., 2003). In the cytoplasm, the pre-miRNA is released from the pre-RISC complex, and the molecule is cut by Dicer (endonuclease RNase III). These processes lead to the production of mature single strand miRNA (Hammond et al., 2000). The mature miRNA interacts with complementary sequence situated within the 3′ untranslated region (3′UTR) of target mRNA, and these base-pairings induce degradation and/or the translational inhibition of its target mRNAs (Bartel, 2009). This opens the possibility of monitoring of mature miRNA action by transfecting reporter transgenes harboring complimentary sequences of miRNAs in their 3′UTR *in vivo* in animals as well as *in vitro* in cells. As miRNAs are involved in controlling bodily development and differentiation, monitoring of miRNA action will help understand the physiological roles of miRNAs in the spatial and the temporal development. Also, understanding miRNA action might lead to the roles of miRNA action in neurological or muscular disorders as well as cancer, autoimmunity, inflammation, and other diseases (Kim, 2005; Krol et al., 2010).

MiR-124 is a well-known regulator responsible for differentiation of progenitor cells to mature neurons. This miRNA is increased during the embryonic stage followed by stable expression in the post-natal period (Krichevsky et al., 2003). The down-regulation of RE1 silencing transcription factor (REST) inhibiting the expression of neuronal genes in non-neuronal cells leads to induce miR-124 expression in neural progenitor cells. This miR-124 degrades small C-terminal domain phosphatase 1 (SCP1) which is a protein of anti-neural function (Conaco et al., 2006; Visvanathan et al., 2007). In neurogenesis, miR-124 down-regulates polypyrimidine tract binding protein 1 (PTBP1) and Sox9 in the subventricular zone (SVZ) (Makeyev et al., 2007; Cheng et al., 2009). Knockdown of endogenous miR-124 maintains purified SVZ stem cells as dividing precursors, whereas ectopic expression leads to precocious and increased neuron formation formation (Cheng et al., 2009; Åkerblom et al., 2012).

Measurements of miRNA expression by Northern blot, real-time PCR, microarray, and *in situ* hybridization are all invasive and used solely for *ex vivo* studies. However, these conventional methods assessing only the amount of miRNA in the fixed time point cannot provide functional action of miRNA over time. To evaluate the miRNA biogenesis and action non-invasively in living animals, *in vivo* optical imaging system has been popularly used especially for mature miRNA action. This optical reporter-based strategy to visualize miRNA action as well as biogenesis was mainly performed using reporter-transfected stem/progenitor cell grafts (Oh et al., 2013). If a reporter gene containing the repeated complementary target sequences of a miRNA is stably incorporated in embryo or adult animal as a form of transgenic animal, this reporter transcript will interact with endogenous miRNAs, leading to the reduction of the reporter propensity and activity due to the reporter mRNA cleavage or translational inhibition. Thus, the endogenous miRNA action can be simply visualized *in vivo* by fluorescence or bioluminescence imaging using fluorescent proteins or luciferases.

Accumulating evidences using miRNA binding sequence-engineered fluorescence reporter have been made to visualize dynamic changes of miRNA level. The transgenic *Drosophila* which contains two copies of perfectly complementary sequence to *bantam* miRNA in the downstream of EGFP reporter was reported as the first fluorescence reporter for *in vivo* (Brennecke et al., 2003). In addition, dual fluorescence reporter containing perfect target sequence for miR-430, miR-1, and miR-133 was engineered to monitor the miRNA expression pattern in the zebrafish embryo or mouse embryos (De Pietri Tonelli et al., 2006; Giraldez et al., 2006; Mishima et al., 2009). The *lacZ* reporter mouse model was designed for detection of the presence of specific miRNAs (Mansfield et al., 2004).

Fluorescence imaging has high background auto-fluorescence and limited tissue penetration *in vivo* but bioluminescence has almost absent background with much higher signal-to-background ratio, better sensitivity and relatively deeper penetration (Kim et al., 2009; Dong et al., 2013; Oh et al., 2013).

Here, we produced a miR-124 reporter transgenic mouse containing miR-124-sensing luciferase reporter gene for tracing the miR-124 action. The luciferase signal of miR-124 reporter gene was significantly decreased by induction of exogenous miR-124 *in vitro*. *In vivo* bioluminescence images were correlated with miR-124 expression on real-time PCR in brain developmental process and while luciferase was expressed variably in the major organs of transgenic mice, luciferase activity of the brain was correlated inversely with endogenous miR-124 expression. Initially high and later dramatic decrease of the bioluminescence in the brain of these transgenic mice are proposed to represent the action of endogenous mature miRNA, i.e., miR-124 in neurons.

## Materials and Methods

### Gene Constructs

The lentiviral vector was derived from pMSCV-ffLuc-pIRES2-Thy1.1 (Rabinovich et al., 2008). The eﬄuc-eGFP-miR-124_3 × PT vector was modified by inserting eGFP into Thy1.1 between BamHI and BglII (Clontech, Mountain View, CA, USA) and 3 tandem repeats of miR-124 perfect target sequences (TTAAGGCACGCGGTGAATGCC) into XhoI site. Viral vector for miR-124 overexpression experiment was derived from pSMPUW-miR-GFP/Puro Lentiviral Expression Vector (Cell Biolabs, San Diego, CA, USA). MiR-124 sequence was obtained from miR-124 precursor sequence including the 100 base flank sequences on both ends of the stem loop from mouse genomic DNA by PCR and clone the blunt-end PCR fragment into the PshA I site of the expression vector. Primers were as follows: forward 5′-tcgaggattcgactgtcctccctctcttccatc-3′, and reverse 5′-tcgagctagcgacttgtactgtgggcgcctgcag-3′. All constructs were verified by sequencing.

### Cell Culture and Transfection

HeLa cell lines were cultured in Dulbecco’s modified Eagle’s medium (DMEM, Welgene, Korea) containing 10% fetal bovine serum (FBS; Invitrogen, Carlsbad, CA, USA) and 1% Antibiotics-antimycotics (Invitrogen, Carlsbad, CA, USA). Primary cortical neurons were isolated from the cortex of E16 transgenic mouse. The primary neurons were dissociated by 0.25% trypsin and plated onto 6 well coated with 1 mg/mL poly-L-lysine. Cortical neurons were grown in DMEM supplemented with N-2 (Invitrogen, Carlsbad, CA, USA), 0.3% Antibiotics-antimycotics and 0.3% FBS in 5% CO_2_ incubator for DIV 10. The cells were transiently transfected with 50 nM scramble, precursor miR-124 or miR-124 inhibitor (Ambion, Austin, TX, USA) using Lipofectamin 2000 (Invitrogen, New York, NY, USA). The miR-124 inhibitor is chemically modified single-stranded RNA molecule to bind endogenous miRNA-124 and enable miRNA functional analysis by down-regulation of miRNA activity.

### Luciferase Assay

Cells were rinsed with phosphate-buffered saline (PBS, pH 7.4), reporter lysis buffer was added, and freeze-thaw cycle was performed to ensure complete lysis. Luciferase activities were measured by GloMax^®^ 96 Microplate Luminometer (Promega, Fitchburg, WI, USA) according to the manufacturer’s instructions of luciferase assay system (Promega, Fitchburg, WI, USA).

### Animals

To generate transgenic (Tg) mouse, we cut the eﬄuc-eGFP-miR-124 3 × PT construct with NdeI and SalI, and ∼4.9 kb transgene which included the 5′UTR and 3′UTR that was obtained from vector. Purified transgene fragment was microinjected into the pronuclei of fertilized C57BL/6N mouse embryos by Macrogen Inc. (Seoul, Korea). Sixty-eight animals were screened and two founders were identified to have the integrated transgene by genotyping and luciferase activity. For genotyping, isolated genomic DNA was tested by PCR analysis using specific primers: forward 5′-GTGAGCAAGAAGGGCCTGCAGAA-3′ and reverse 5′-CCGCTGGTTCACGCCCAGGGTC-3′. Founders were backcrossed to C57BL/6N mouse or intercrossed.

### *In vivo* and *Ex vivo* Luciferase Bioluminescence Imaging

All of the animal experimental procedures were approved by Seoul National University Hospital Institutional Animal Care and Use Committee [Approval number: 15-0050-C1A0(1)] and performed in accordance with the guideline from the institute. This institute cares animals in accordance with Association for Assessment and Accreditation of Laboratory Animal Care International (AAALAC International). All To the animals, we administered 3 mg/0.1 mL/mouse luciferase substrate luciferin (Caliper, Newton, MA, USA) intraperitoneally, and the animals were sedated 15 mins after D-luciferin injection with 2.5% isoflurane in O_2_ gas flowing through isoflurane/oxygen main tank at a flow rate of 1.5 L/min. The bioluminescence images were acquired for 10–600 s using IVIS-100 imaging system equipped with a CCD camera (Xenogen, Alameda, CA, USA).

For *ex vivo* imaging of organs, the embryo and organs were collected from anesthetized mouse after scanning the whole body. The organs were exposed for 10 s in order to acquire bioluminescence imaging. All image acquisition and data analysis was conducted using Living image software (Xenogen, Alameda, CA, USA).

### MiRNA and mRNA Real-Time PCR

Total RNA was extracted from isolated organs with TRIzol (Invitrogen, Carlsbad, CA, USA) according to the manufacturer’s instruction. 10 ng of RNA was processed for cDNA synthesis using specific Taqman primer (Applied Biosystems, Carlsbad, CA, USA) and M-MLV reverse transcriptase (Invitrogen, Carlsbad, CA, USA). cDNA was then amplified on the 7500 real-time PCR system (Applied Biosystems, Carlsbad, CA, USA), employing the ΔΔCt method with Taqman assays to quantify the relative expression level. MiRNA and mRNA level measurements were done in triplicate for each experiment. The following Taqman assays were used in this study: miR-124 (001182), and U6 (001973).

### Luciferase and GFP Immunohistochemistry

Isolated organs were placed in 4% PFA for 48 h, and then made into paraffin embedded tissue blocks. 4 μm paraffin sections were deparaffinized by oven heating and by immersion in xylene. After dehydration through graded alcohols to water, slides were boiled in 0.01 M citrate buffer (pH 6.0) for 10 min then maintained at a sub-boiling temperature for 20 min to retrieve antigen, and further immersed in 1% H_2_O_2_ in methanol for 30 min. Sections were incubated for 30 min with TBS containing 0.5% Triton X-100 (TBST), before blocking each sections with blocking solution (5% bovine serum albumin in TBST) for 30 min at room temperature. Sections were incubated at 4°C overnight with primary antibody against goat anti-luciferase or GFP (Abcam, Cambridge, UK). After washing with TBST, antibody staining was revealed using the species-specific Alexa Flour 488 conjugated secondary antibody. 1 μM 4′.6-diamidino-2-phenylindole (DAPI, Invitrogen, Carlsbad, CA, USA) was used for counterstaining. Confocal microscopic observation was performed using Carl Zeiss LSM510.

### Immunocytochemistry

Cells were washed twice with PBS, and then fixed for 20 min with 4% paraformaldehyde (PFA). After fixation, the cells were incubated for 30 min in PBS containing 0.1% Triton X-100 for permeabilization, and then blocked by incubating the cells in blocking buffer (3% normal goat serum, 2% BSA and 3% Triton-X 100 in PBS) for 1 h at room temperature. Cells were then incubated for overnight at 4°C in PBS containing primary antibodies against rabbit anti-MAP2 (Sigma, St. Louis, MO, USA) and mouse anti-GFAP (Cell Signaling Technology, Danvers, MA, USA). After washing with PBS, cells were incubated with Alexa Flour 488- or 555-conjugated secondary antibody (Invitrogen, Carlsbad, CA, USA) and 1 μM DAPI (Invitrogen, Carlsbad, CA, USA) at room temperature. Stained cells were observed using a confocal microscopy (Carl Zeiss LSM510, Jena, Germany).

### Statistical Analysis

Data are expressed as the means ± SD values. One-way ANOVA and Student’s *t*-test were used to determine statistical significance where *p* < 0.05 was considered to be significant.

## Results

### Production of miR-124 Action Reporter Vector and Its Validation *In vitro*

**FIGURE 1:**
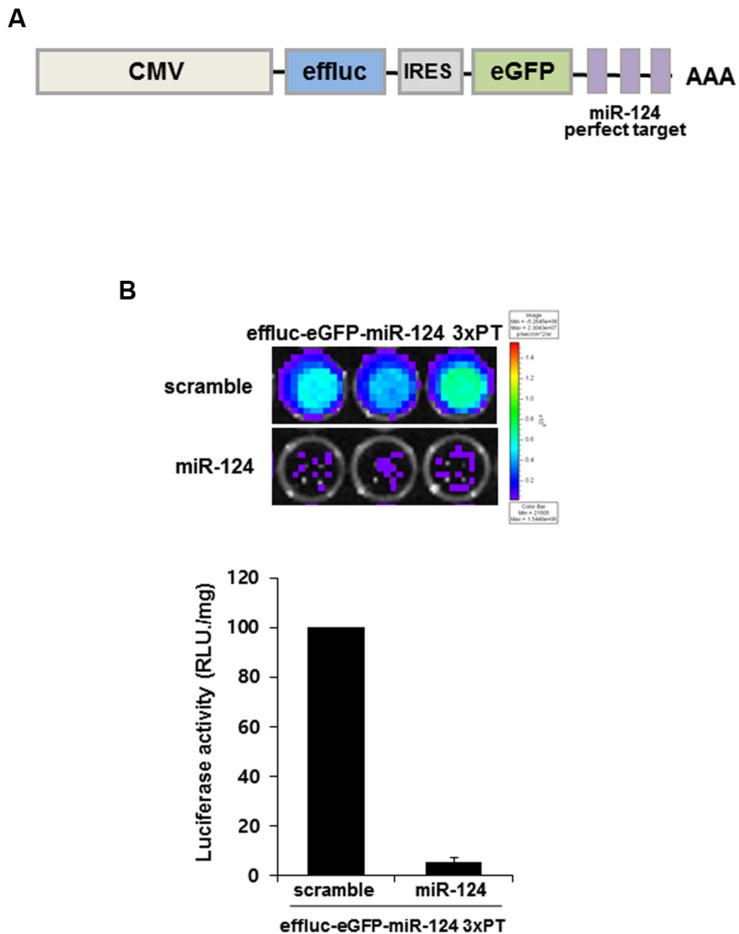
**MiR-124 action reporter vector and its validation. (A)** Schematic diagram of miR-124 reporter transgene of lentiviral vector. The lentiviral construct consists of CMV promoter, eﬄuc, IRES, and 3 × PT (triple perfect targets) including three repeat perfect target sequences complementary to mature miR-124. **(B)** Luminescence produced after 24 h of transient transfection of scramble or miR-124 showed working of eﬄuc in the eﬄuc-eGFP-miR-124_3 × PT transfected HeLa cells. Luciferase activity (photons/second) was quantified using Living image software. Data are represented by the means ± SEM (*n* = 3).

In order to produce reporter construct for monitoring miR-124 action (previous ID: miR-124a), we generated a construct containing three copies of a miR-124-perfectly matched sequence (miR-124_3 × PT) with the enhanced firefly luciferase (eﬄuc) and enhanced GFP (eGFP) reporter genes in its 3′UTR. Using IRES (internal ribosome entry site) lentiviral vector, eﬄuc-eGFP-miR-124_3 × PT construct co-expressed eﬄuc and GFP genes from a single mRNA transcript. This allowed miR-124 to suppress the expression of two reporter proteins (**Figure 1A**). In order to validate the constructs, HeLa cells were co-transfected with reporter construct and synthetic scramble or miR-124 precursors. We found that miR-124 overexpression significantly affected luciferase activity of eﬄuc-eGFP-miR-124_3 × PT-infected cells by luciferase assay (5.37 ± 1.85%, *p* < 0.01, **Figure 1B**). Eﬄuc-eGFP-miR-124_3 × PT construct was considered suitable for monitoring miR-124 action as well as biogenesis, hence generation of a new transgenic mouse for *in vivo* monitoring of miR-124 using this construct was further investigated.

### Generation of miR-124 Reporter-Harboring Transgenic Mice

**FIGURE 2:**
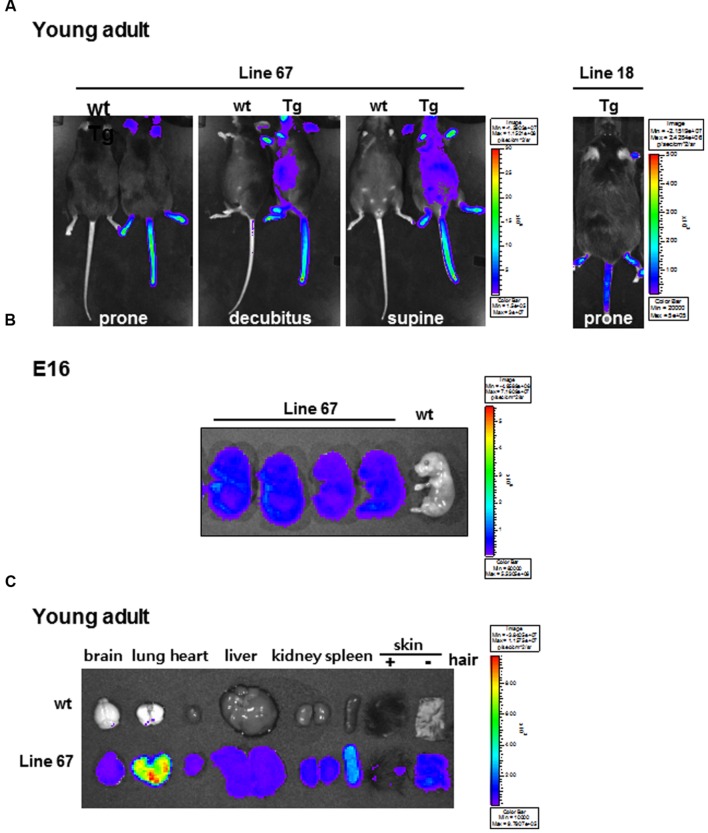
**Bioluminescence images of candidate miR-124 reporter-expressing transgenic mice. (A)** Whole body bioluminescence images of wt and transgenic mice harboring eﬄuc-eGFP-miR-124_3 × PT acquired with D-luciferin injection. The acquisition time of line 67 (left) and line 18 (right) was 10 s and 600 s, respectively. **(B)** *Ex vivo* images of eﬄuc reporter activity of fetuses of wt mouse and transgenic mice (line 67) at E16 period. **(C)** *Ex vivo* images of major organs excised from a wt and a transgenic mouse (line 67).

Transgenic mice were produced by pronuclear injection of eﬄuc-eGFP-miR-124_3 × PT gene using the CMV promoter to drive the expression reporter transgenes in high amount. We tested whether the transgenic mouse was able to carry out stable germ-line transmission and inheritance. After D-luciferin injection, we found two positive transgenic mice (line 18 and line 67) among 11 founders emitting bioluminescence signals. The origin mouse of transgenic strain C57BL/6 mouse (wild type: wt) was used as negative control (**Figure 2A**). High bioluminescence signal was observed in the areas of hairless skin such as snout, ears, tail, and feet. In whole body imaging, the bioluminescence was greater in line 67 than in line 18 (**Figure 2A**). As shown in **Figure 2B**, the bioluminescence was prominent throughout the whole body of E16. Luciferase signals were also observed in the major isolated organs of transgenic mice, compared to those of wt mouse, which revealed that CMV promoter let the transgenes express in a variety of mouse tissues and cells (**Figure 2C**). Interestingly, in young adults, lung activity was the most prominent and removal of hairs from the skin made skins shine (**Figure 2C**). Line 67 was used for further experiment of miR-124 imaging. In *ex vivo* measurement of isolated organs of line 18, luciferase signals were shown only in the skin but not in the major organs (data not shown). There was no difference in miR-124 expression between wt and transgenic mice in their various areas of the brains on *in situ* hybridization analysis (**Supplementary Figure S1**). We concluded that a line of transgenic mouse containing eﬄuc-eGFP-miR-124_3 × PT transgene was established.

### Expression of miR-124 and Luciferase in the Brain and the Other Organs of Transgenic Mice

**FIGURE 3:**
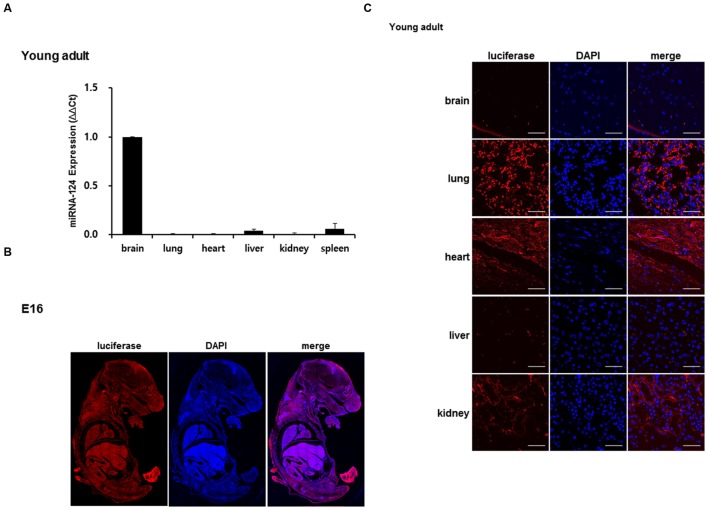
**Validation of luciferase expression of a miR-124 reporter transgenic mouse. (A)** In a young adult mouse, quantitative RT-PCR of tissue RNAs of brain and major organs revealed prominent expression of miR-124 normalized to that of U6. The expression of other organs was represented using a fold increase relative to the value 1 of the brain [means ± SD (*n* = 3)]. **(B)** At E16 period of a transgenic mouse, luciferase transgene expressed as shown and counterstained by DAPI on whole body immunohistochemistry **(C)**. Tissue sections of major organs of a transgenic mouse showed distribution of cells counterstained with DAPI (blue) and propensity of luciferase staining (red). Compared with highest lung and moderate heart/kidney activities, luciferase of brain and liver was scarce on confocal microscopy at 400× magnification. The scale bars represent 50 μm.

Real-time PCR was done with the total RNA of the brain and other organs to determine the expression of miR-124. In young adult mouse, miR-124 expression was approximately 100-fold higher in the brain, compared to the other organs (lung, heart, liver, kidney, and spleen) (**Figure 3A**). Luciferase was detected using immunohistochemistry with transgenic embryo sections and with major organs of the young adult mouse (**Figures 3B,C**). In the whole body of E16, luciferase transgene was expressed throughout the entire body (**Figure 3B**). The distribution was uneven, but due to the difference of cell density between organs, the relative density or rarity could not be documented. In the young adults, luciferase counter-stained with DAPI showed evenly distributed immunoreactivity in major organs, among which the lungs showed the highest expression (**Figure 3C**). As we expected, the luciferase was rarely seen in the brain and was also barely seen in the liver in young adult mice. This *ex vivo* study showed that luciferase was expressed in other organs with the least miR-124 expression and luciferase was suppressed in the brain (and liver) in young adult periods.

### Comparison of Luciferase Bioluminescence and miR-124 Expression in the Brain during Development

**FIGURE 4:**
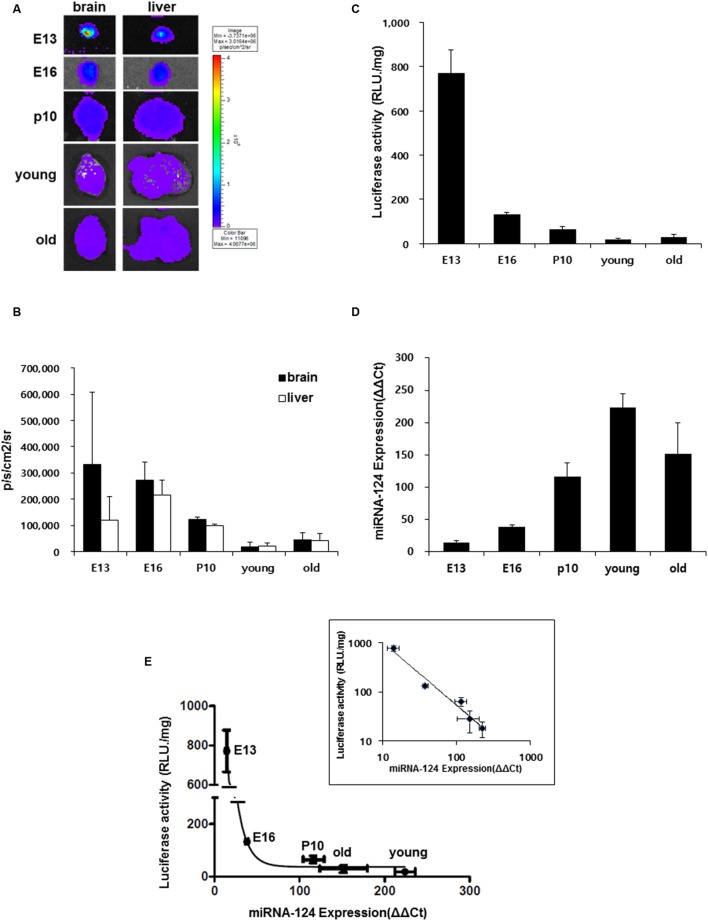
**Developmental stage-dependent miR-124 expression regulates luciferase signal. (A)** *Ex vivo* bioluminescence images of brain and liver of a transgenic mouse along the bodily development *in utero* and after birth (E13 *n* = 6, E16 *n* = 6, P10 *n* = 3, young *n* = 3, and old *n* = 4); 3∼5 month old (young), 16∼24 month old (old). **(B)** Quantified results of these *ex vivo* images. Average counts of the brain and the liver was shown as photons/sec/cm2. The brain luciferase was maximal at E13 and decreased to the minimum at young adult period and the liver luciferase was maximal at E16 and minimum at young adult period. **(C)** Luciferase activity (RLU/mg) of brain lysate in each developmental stage. **(D)** Quantitative real-time RT-PCR results of mir-124 over U6 of the brain lysates in each developmental stage. Data are shown as means ± SD. RLU/mg means relative light units per mg of protein of the lysate. **(E)** Relationship between miR-124 expression and luciferase activity of brain lysates which showed inverse relation. After log-log transform, the relation was inverse-proportional (inset). Whisker of each point represents S.D.’s of miR-124 expression (abscissa) and luciferase activity (ordinate).

The pattern of miR-124 expression and luciferase reporter bioluminescence was compared using brain tissues in each period of E13, E16, P10, young and old adult with *ex vivo* luminescence imaging. At E13 period, the bioluminescence of the brain was higher than that of the liver on luciferase imaging but it decreased at E16, further at P10 and it was the lowest in the young adult period (**Figures 4A,B**). In contrast, bioluminescence of the liver was the highest at E16 and decreased at P10 and it was the lowest in the young adult period (**Figures 4A,B**).

Using brain lysates of each periods of E13, E16, P10, young and old adult, on luciferase activity assay, luciferase activity was highest at E13 and 17.1 ± 1.2% at E16 compared with at E13 and decreased further in the young adult period (**Figure 4C**). Using the same lysates, on quantitative real-time PCR for miR-124, miR-124 concentration was lowest at E13 and increased continuously via E16 to the young adult period (**Figure 4D**). When the relationship between *ex vivo* concentration of miR-124 and luciferase activity was plotted, it was found to be inverse-log-log-proportional (**Figure 4E**).

### Cortical Neurons Extracted from the Transgenic Mouse Responded to Transfected miR-124

**FIGURE 5:**
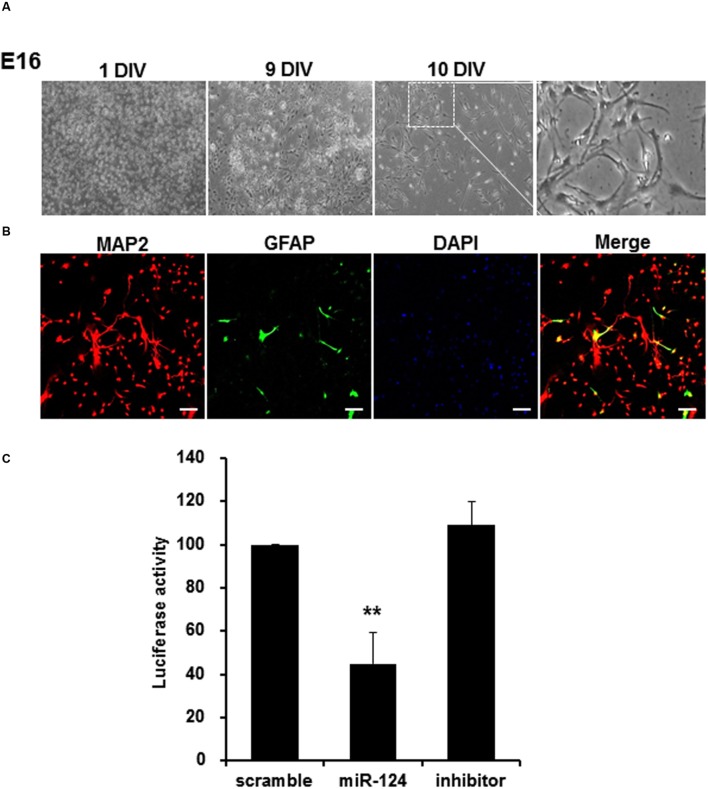
**MiR-124 reduces luciferase activity in the cortical neurons extracted and cultured from transgenic mouse. (A)** Cortical neurons from E16 of transgenic mouse were cultured for 10 days *in vitro* (DIV). Images were displayed with phase-contrast microscope using 4× or 100× magnification (inset). The scale bars represent 200 μm (low-scaled panel) and 50 μm (magnified panel). **(B)** Immunocytochemistry with anti-MAP2 (microtubule-associated protein-2, red, and labeling neurons) and anti-GFAP (Glial fibrillary acid protein, green, and labeling astrocytes) antibodies counterstained with DAPI were shown with confocal microscope using a 100× magnification. The scale bars represent 100 μm. 95% were MAP2 positive and the other 5% were GFAP positive. **(C)** Primary cultured cortical neurons which were transfected with scramble, miR-124, or miR-124 inhibitor at DIV 10, showed reduction of luciferase activity only with miR-124. The results were normalized as percent ratios to the scramble treatment. Data were represented by means ± SD (*n* = 6, ^∗∗^*p* < 0.01).

Cortical neurons extracted from E16 transgenic mice were cultured *in vitro*, and confirmed to have transgene genotypes (data not shown). Under phase contrast microscope, these neurons showed neuronal features along with lapsing *in vitro* days until 10 days *in vitro* (DIV) (**Figure 5A**). On immunocytochemistry using antibodies against MAP2 and GFAP, which labeled mature neurons and astrocytes, respectively, the proportion of primary cortical neurons was 94.9 ± 2.5% (**Figure 5B**). When these cortical neurons were transfected with scramble, miR-124 or miR-124 inhibitor for 24 h, miR-124 transfection alone significantly decreased the luciferase activity by 55.3%, compared to scramble transfection (**Figure 5C**). MiR-124 inhibitor did not change (increase) luciferase activity.

## Discussion

We produced a reporter transgenic mouse model harboring eﬄuc-eGFP-miR-124_3 × PT transgene to visualize miR-124 action which plays an important role in neurogenesis under physiological conditions. MiR-124 suppresses expression of target mRNAs such as an anti-neural factor SCP1, transcription factors Sox9, Notch ligand Jagged1, and the BAF53a subunit of the chromatin-remodeling complex, and induces neurogenesis during the neural development (Visvanathan et al., 2007; Cheng et al., 2009; Yoo et al., 2009; Liu et al., 2011). Another target of miR-124 is PTBP1 which has been recognized as a global repressor of alternative pre-mRNA splicing in non-neuronal cells. The reduced expression of PTBP1 promotes neuron-specific alternative splicing of PTBP2, which is sufficient to decide the cell fate toward the neuronal lineage (Makeyev et al., 2007; Xue et al., 2013).

In order to design a construct that gets modulated by miR-124, we inserted three repeats of perfectly matching complementary sequences of mature miR-124 to the downstream of reporter gene under the CMV promoter (**Figure 1A**). The luciferase gene was used as a reporter gene due to high signal to background ratios and relatively high tissue penetration (Kim et al., 2009; Oh et al., 2013). The transgenic mice containing eﬄuc-eGFP-miR-124_3 × PT transgene showed the bioluminescence signals in major organs including the skin by the CMV promoter, which is one of the strongest known promoters (**Figures 2A,C**). The luciferase activity of glial cells, which do not express miR-124, may contribute to the minimal bioluminescence of the brain during young adult period (**Figure 4A**) when the expression of miR-124 was maximal in brain mainly in neurons (**Figures 3A** and **4D**). However, green fluorescence was not observed in any organs of transgenic mice, though we could find GFP immunoreactivities in the tissues slices of transgenic mice which should have expressed after the IRES segment (**Supplementary Figure S2**, Ibrahimi et al., 2009). GFP immunoreactivities in major organs of transgenic mouse were consistent with luciferase immunoreactivities (**Supplementary Figure S2**), which were again consistent with bioluminescence imaging *ex vivo* (**Figure 2C**). The observed action of miR-124 on the 3′UTR of transgene transcript in this study proves that our transcript worked to produce functionally active luciferase protein under the expected regulation by endogenous miR-124 (**Figure 4E**). We also observed bioluminescence of their embryos to confirm integration and stable germ line transmission of the transgene (**Figure 2B**).

On real-time PCR of miR-124 and immunohistochemical analysis of luciferase, young adult transgenic mouse showed high expression of miR-124 and low expression of luciferase in the brain (**Figures 3A,C**). It has also been reported that miR-124 is expressed specifically in the nervous system (Conaco et al., 2006; Makeyev et al., 2007; Visvanathan et al., 2007; Cheng et al., 2009) and starts to increase in the brain at E10 period of mice (Krichevsky et al., 2003; Farrell et al., 2011). In our study, miR-124 expression reached the maximum in young adult period and this increase was represented inversely well with measurement of luciferase activity in tissue lysates (**Figures 4C,D**).

In our eﬄuc transgenic mouse, once we extracted the brain to evade the high background luminescence of the skin, we could easily visualize the intensity and distribution of luminescence in the entire brain (**Figure 4A**). We observed changing expressions of miR-124 in their brain of each developmental stage by bioluminescence imaging, lysate luciferase assay and real-time PCR. Interestingly, the amount of mature miR-124 transcript and quantified luciferase activity on lysate luciferase assay *ex vivo* were inversely log-log proportional. As changing intensities of *ex vivo* bioluminescence imaging of **Figure 4A** almost copied the changes of lysate luciferase assay, *ex vivo* bioluminescence imaging predicted the amount of miR-124 transcript of the brain. We propose that *in vivo* bioluminescence imaging can also predict the amount of miR-124 transcript *in vivo* without sacrifice of the animal. In fact, *in vitro* real-time PCR yields only the amount of miR-124 but not the action of mature miR-124 *in vivo* and thus, we could say that luciferase bioluminescence or activity represent the miR-124 action *in situ* as well as the amount of miR-124.

Similar to the results reported here, neurogenesis occurs at E9 to E10 and peaks at E14 to E15 in most parts of the brain, and gradually decreases until birth (Götz and Huttner, 2005; Rowitch and Kriegstein, 2010; Díaz et al., 2014). MiR-124 induces neuronal differentiation and inhibits glial differentiation of embryonic stem cells during this transition of neurogenesis (Conaco et al., 2006; Makeyev et al., 2007; Visvanathan et al., 2007; Åkerblom et al., 2012). This changing pattern of neurogenesis associated with miR-124 is further supported by our results, demonstrating an increase of the amount of miR-124 at E13 via E16 to the peak at young adult period (**Figure 4D**). What, we have shown more in this investigation is that by observing miR-124 action using luciferase bioluminescence, miR-124 really acted as mature microRNA to suppress its target proteins including our transgenes.

We also found that the organs with lower miR-124 expression than the brain showed variable levels of luciferase on immunohistochemistry at E16 and in young adult period (**Figures 3B,C**). The difference of luciferase expression among the organs was assumed to be due to the different levels of methylation of CMV promoter in each organ (Kong et al., 2009). In a transgenic pig model expressing GFP driven by a CMV promoter which was used to analyze the correlation between the copy number and the promoter methylation, they demonstrated that GFP mRNA amounts were almost 20-fold higher in certain organs than those in other organs (Kong et al., 2009). In our transgenic mouse model of line 67, the lungs expressed the luciferase the highest amount and heart and kidneys also expressed luciferase in high amounts. But, in another model of line 18, all the internal organs expressed almost no luciferase which could either mean silencing of CMV promoter or suppression of luciferase by high miR-124 in these organs. Considering absent expression of miR-124 in other organs (**Figure 3A**), CMV promoter of the transgenes would have been silenced in line 18 as well as in the other failed cases of transgenic mouse production.

This transgenic mouse model can be a source for stem/progenitor cells for transplantation. We extracted cortical neurons from this transgenic mouse at E16 period and cultured these cells. After administration of miR-124 to these cells, luciferase of these cells was suppressed and thus, we could know that the luciferase transgene mRNAs were under the control of the exogenous miR-124 (**Figure 5**). We think that transgenic mouse and derived cells are under good control of miR-124 and luciferase transgene would work well to report the action of mature miR-124. This implies that the lower luciferase expression of the brain at E16 than at E13 but yet higher than adult periods is sufficient to show further suppression of reporter transgene expression by the extraneous abundant miR-124. We propose that comparison of quantified bioluminescence after directly injected or targeted delivery of miR-124 enable the therapeutic efficacy of this targeted action using this eﬄuc-eGFP-miR-124_3 × PT Tg mice. However, for quantitative *in vivo* imaging, considering the ubiquitous expression of reporter transgenes due to CMV promoter especially in skin which can obliterate brain imaging, another transgenic mouse with a brain-specific promoter might be generated as an improved alternative. And the thickness of brain skull affects quantitative *in vivo* bioluminescence imaging due to low rate of photonic emissions under the skull. Thus, development of ultrasensitive luciferase modification or new bioluminescence imaging device capable of three-dimensional bioluminescence tomography is essential to obtain high quality image signals.

The transgenic mice bearing the eﬄuc-eGFP-miR-124_3 × PT as well as transgenic mice bearing GFP-miR-124_4 × PT of Akerblom’s group employ a signal-off system (Åkerblom et al., 2012). If our or their transgenic mice will be the source of donor stem/progenitor cells in cell transplantation experiments, the difficulty of discrimination between cell death or transgene silencing and signal-off when, we were trying to determine whether the stem cells differentiate into the neurons after implantation. To overcome this problem, signal-on system using a fluorescent nanoparticle-based molecular beacon with surface-bound nucleic acids (Hwang et al., 2010) was proposed to monitor microRNA action *in vivo* and thus transfection of the stem/progenitor cells using molecular beacons will be necessary for further experiment (Tyagi and Kramer, 1996).

Åkerblom et al. (2012) have demonstrated a miR-124 sensor transgenic mouse containing GFP-miR-124 4 × PT and used this transgenic model to show that miR-124 overexpression induces neuronal differentiation from neural stem cells, promoting neurogenesis in SVZ. They also showed that the inhibition of miR-124 via sponge expression blocks neurogenesis, leading to the development of ectopic cells with astrocyte characteristics in the olfactory bulb (Åkerblom et al., 2012). We propose that unlike miR-124-sensing GFP transgenic mice, we could quantify luciferase activity using bioluminescence imaging which was inversely correlated with miR-124 level in the brain. This model mouse can be used for monitoring dynamics of miR-124 repeatedly *in vivo* and after the last imaging, confirmation of the results are possible using *ex vivo* bioluminescence imaging and the classical luciferase assay and RT-PCR of the lysates and *in situ* hybridization and immunohistochemistry. This model can also be used for proving the success of miRNA or siRNA delivery to the neurons by observing the action of miRNA/siRNA against miR-124 targets *in vivo*.

## Conclusion

We produced a transgenic mouse and proved that luciferase transgenes in the brain as well as the other organs are under endogenous miR-124 control using real-time RT-PCR, luciferase activity assay using lysates, and luciferase imaging *ex vivo* as well as *in vivo* and finally proven by immunohistochemistry. Most importantly, *in vivo* action of miR-124 was represented by luciferase bioluminescence imaging associated with quantification. Using this miR-124 reporter mouse, miR-124 was expressed in a changing fashion during development in utero and after birth, which recapitulated previous studies about the role of miR-124 in neurogenesis. This animal model can be used to investigate the role of miR-124 in various physiological and pathologic conditions. The final outcome of successful delivery of therapeutic miRNA/siRNA can now be evaluated non-invasively to demonstrate the biodistribution of its action (not just the delivery) following systemic or topical delivery of miRNA by observing the action of these therapeutic nucleic acids in transgenic animals.

In our eﬄuc transgenic mouse, we extracted the mouse brain to minimize the high background signal from the skull, and we could easily visualize the signal intensity and distribution of bioluminescence in the entire brain (**Figure 4A**). Indeed, unlike fluorescence imaging that has intrinsic limitation such as high non-specific background due to the requirement of external light source, bioluminescence imaging that does not use excitation light can offer better imaging quality. Nevertheless, the thickness of brain skull may hamper the good penetration of emitted light from luciferase-luciferin reaction, decreasing signal sensitivity. Thus, development of ultrasensitive luciferase modification or new bioluminescence imaging device capable of three-dimensional bioluminescence tomography is essential to obtain high quality image signals.

## Author Contributions

DSL and DWH designed the study and revised the manuscript. YC wrote the main manuscript text and prepared figures. JYK performed **Supplementary Figure S1**. WS and MYK discussed the results. All authors reviewed the manuscript.

## Conflict of Interest Statement

The authors declare that the research was conducted in the absence of any commercial or financial relationships that could be construed as a potential conflict of interest.

## Supplementary Material

FIGURE S1 **MiR-124 was expressed well after reporter insertion in the brain of transgenic mouse at young adult period.** The expression pattern was not different between wt and transgenic mouse in that all the *in situ* histochemical activity resided in the neurons of cortex (CTX), hippocampal cortex (HC), striatum (STR), rostral migratory stream (RMS), and SVZ.

FIGURE S2 **GFP expressions in major organs of transgenic mouse were measured by immunohistochemistry using anti-GFP (green), and counter-stained the sections with DAPI (blue).** GFP immune reactivities in major organs of Tg mouse were compared with luciferase immunoreactives.
