# Supplementary figures and images for: Transgenic Mouse Expressing Optical MicroRNA Reporter for Monitoring MicroRNA-124 Action during Development
Source: Front Mol Neurosci. 2016 Jul 12;9:52. doi: 10.3389/fnmol.2016.00052 (PMC4940420; doi:10.3389/fnmol.2016.00052)

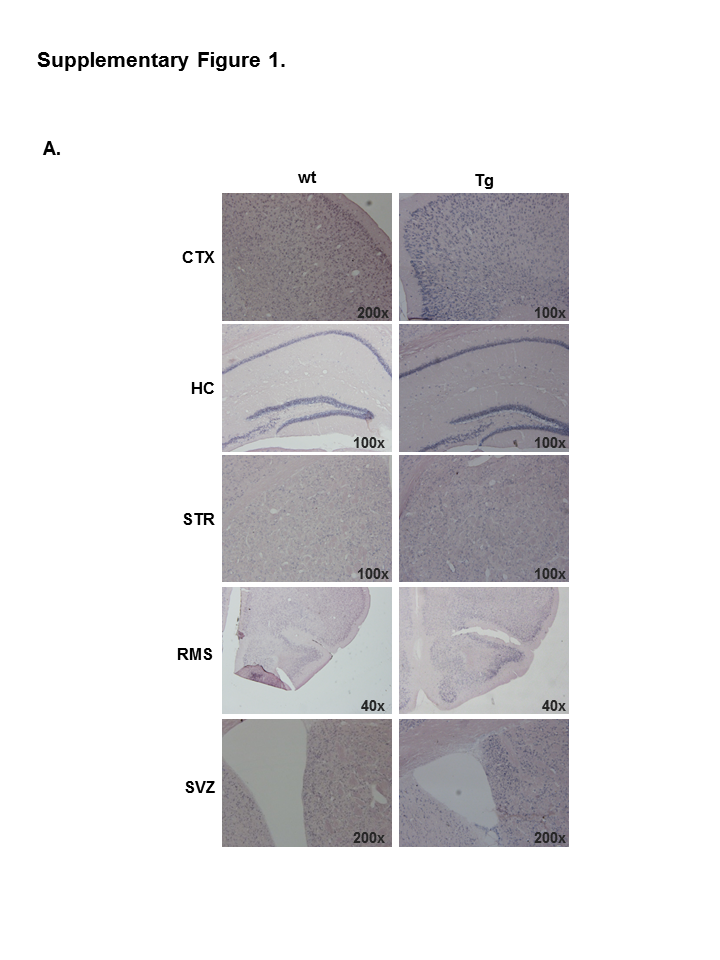

Supplement: FIGURE S1 — MiR-124 was expressed well after reporter insertion in the brain of transgenic mouse at young adult period. The expression pattern was not different between wt and transgenic mouse in that all the in situ histochemical activity resided in the neurons of cortex (CTX), hippocampal cortex (HC), striatum (STR), rostral migratory stream (RMS), and SVZ. [file Image_1.TIF]

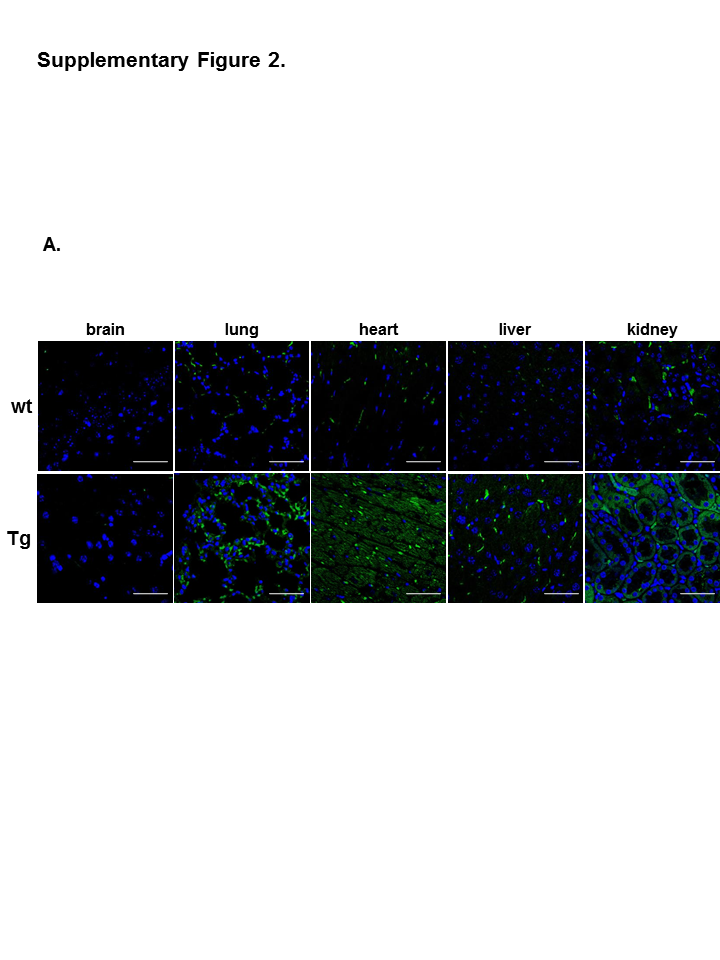

Supplement: FIGURE S2 — GFP expressions in major organs of transgenic mouse were measured by immunohistochemistry using anti-GFP (green), and counter-stained the sections with DAPI (blue). GFP immune reactivities in major organs of Tg mouse were compared with luciferase immunoreactives. [file Image_2.TIF]
